# Management of a patient at high risk of type 2 diabetes

**DOI:** 10.1080/17571472.2016.1203509

**Published:** 2016-07-25

**Authors:** Lara Howells, Ailsa J. McKay, Sufyan Hussain, Azeem Majeed

**Affiliations:** ^a^ Department of Primary Care and Public Health, Imperial College London, London, UK; ^b^ Department of Diabetes, Endocrinology and Metabolism, Imperial College London, London, UK

**Keywords:** Intermediate hyperglycaemia, HbA_1C_, diabetes prevention

## Abstract

Rates of type 2 diabetes mellitus have risen rapidly over the past three to four decades. This article describes a typical patient presenting with intermediate hyperglycaemia in primary care. We suggest the appropriate action to reduce the risk of diabetes developing. Population-level preventive interventions, and adequate recognition and early management of those at risk of developing diabetes, could mitigate the impact of this evolving health epidemic.

## Background

Type 2 diabetes mellitus (T2DM) is a chronic disease associated with reduced quality of life and reduced life expectancy.[1] June 2015 estimates suggest that approximately 3.3 million people in the UK currently have a diabetes diagnosis, equivalent to 6.2% of the population.[2] Rates of T2DM have risen rapidly over the past three to four decades, largely attributed to similar prevalence trends for overweight and obesity, an ageing population and changes to population ethnic composition.[3,4] Ongoing upward trends in risk factors will lead to continued increases in prevalence, projected to reach 9.5% by 2030.[2] The associated costs are high. 23,300 UK deaths were attributed to diabetes in 2010–11. In the same year the direct costs of diabetes to the UK were £8.8 billion, and the indirect costs £13 billion.[2] Population-level preventive interventions, and adequate recognition and early management of those at risk of developing diabetes, could help mitigate the impact of this evolving health epidemic.

## Intermediate hyperglycaemia

**Figure 1.**
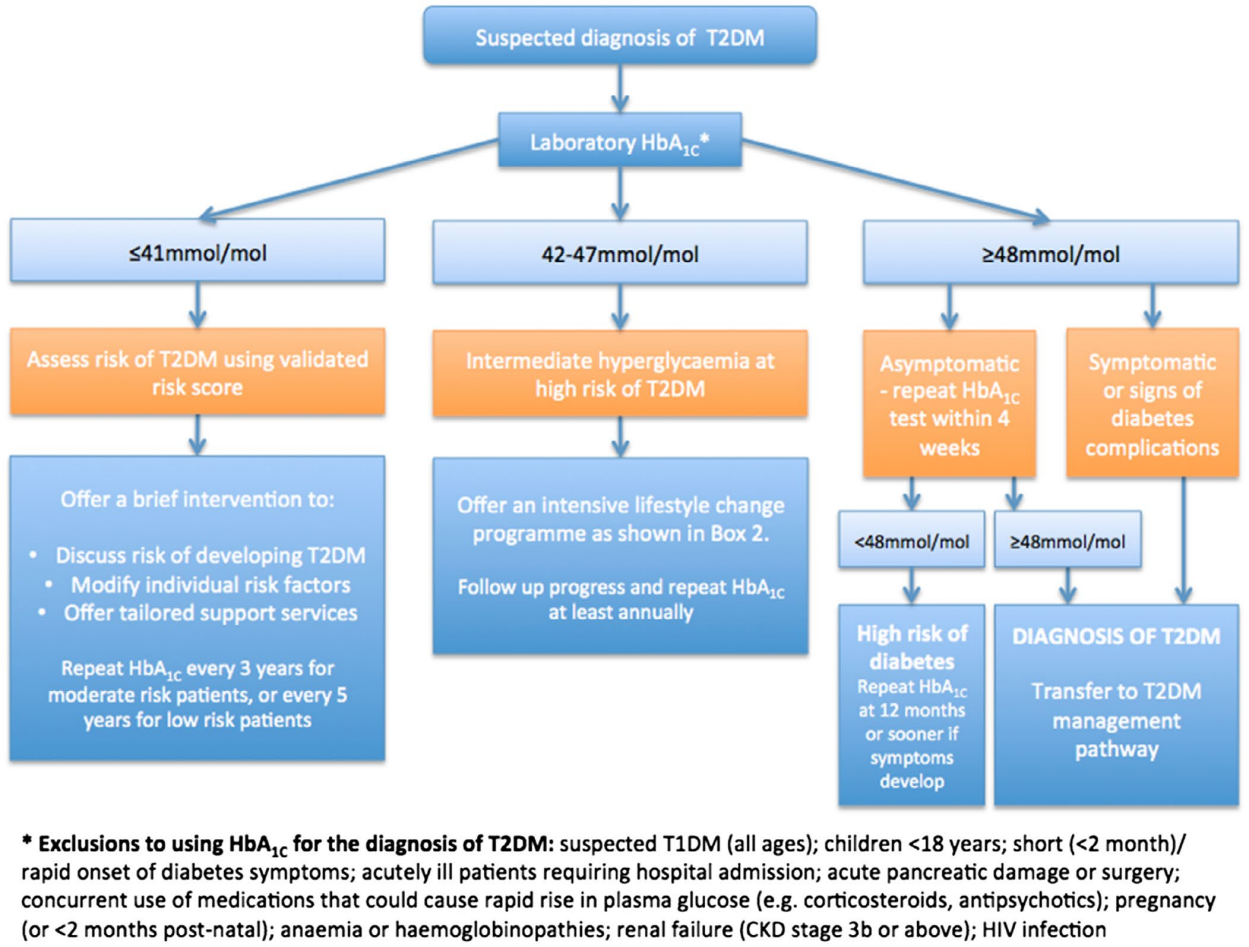
Algorithm for the diagnosis of T2DM, adapted with permission from the ‘Consensus approach to the diagnosis of Type 2 diabetes’.[8]

National Institute for Health and Care Excellence (NICE) guidance suggests that HbA_1c_ (glycated haemoglobin) or fasting blood glucose (FBG) measurement is undertaken when a validated diabetes risk assessment tool produces a high risk score.[5] An HbA_1c_ level between 42 and 47 mmol/mol (6.0–6.4%), or fasting blood glucose measurement of 5.5–6.9 mM – described as intermediate, or non-diabetic, hyperglycaemia – is associated with a relatively high risk of developing T2DM (see Figure 1).[5] In managing a finding of intermediate hyperglycaemia, it is first important to establish that an HbA_1c_ measurement is reliable. It can be unreliable in the presence of haemoglobinopathies and other conditions that modify red blood cell turnover, among other things (see Figure 1). It is not recommended for use in children, young people, or pregnant women. It is also important to note any potential alternative cause of hyperglycaemia, and an understanding of the patient’s risk factors for diabetes and cardiovascular disease should be developed. Risk factors for diabetes include family history, advancing age, overweight/obesity, hypertension, dyslipidaemia, smoking, physical inactivity, ethnicity, and history of gestational diabetes, polycystic ovarian syndrome or pre-existing cardiovascular disease.[6] This list includes all the well-established modifiable cardiovascular risk factors.

## A typical presentation

A typical presentation of intermediate hyperglycaemia is displayed in Box 1.

Box 1. A 48-year-old Caucasian woman consults you. As she was in the target group (40–74 years, and without pre-existing cardiovascular disease), she has recently been invited for an NHS Health Check. At her Health Check, she was found to have an elevated HbA1c value of 44 mmol/mol (6.2%). She was also noted to be overweight. Her other NHS Health Check measurements such as blood pressure, lipids and renal function were normal. She has not previously been known to have a raised HbA1c and there was no family history of diabetes.

In such a case, after the history (incorporating the points mentioned above) has been established, and it is understood that the finding does represent intermediate hyperglycaemia, the patient’s prior knowledge of type 2 diabetes and its risk factors should be established. It is important to ensure the patient understands that diabetes is a chronic disorder of glucose metabolism that requires close monitoring, and is associated with several debilitating macrovascular and microvascular complications. The increased risk of cardiovascular disease associated with intermediate hyperglycaemia should be discussed. As the major modifiable risk factors for cardiovascular disease are largely consistent with those for diabetes, the importance of associated risk reduction can be emphasised.[7] In this particular case, it would be particularly appropriate to explore the patient’s insight and perception of their health related to weight, and to seek to establish whether the patient has made any prior commitment to lifestyle change. The outcome of previous or current attempts to make lifestyle changes should be considered.

## Action to take

The principles of risk reduction should be explained to the patient and all modifiable risk factors for diabetes and cardiovascular disease should be addressed:

*Blood pressure* – Her blood pressure is currently within the reference range; it should be maintained below 140/90 mm Hg and ideally below 130/80 mm Hg.

*Lipid control* – Calculate the patient’s 10-year cardiovascular risk using the QRISK2 algorithm (http://www.qrisk.org). Explain that lifestyle change is the preferred method of lipid control if her 10-year risk of cardiovascular disease is <10%. If her cardiovascular risk is found to be high (i.e. ≥10% 10-year risk), consider prescribing a statin (e.g. atorvastatin 20 mg) in addition to lifestyle modification.[5]

*Smoking cessation* – Offer assistance and referral to a smoking cessation service if she is a smoker.

*Lifestyle interventions* – Where appropriate, offer referral to a local evidence-based quality-assured intensive lifestyle-change programme (see Box 2). The onset of diabetes can be prevented or delayed through weight loss, dietary changes and increased physical activity,[3] but making and sustaining the required necessary lifestyle changes is challenging. Assess her motivation to engage in supported lifestyle modifications, and explore potential barriers. Involvement of a family member, carer or friend for emotional and practical support should be encouraged.[5]

*HbA1c* – Set an HbA_1C_ target.

Box 2. Intensive lifestyle-change programmes offer tailored encouragement to help people:

• Undertake a minimum of 150 min of moderate-intensity exercise per week.
• Gradually lose weight to reach and maintain a healthy BMI (<25 kg/m^2^).
• Increase intake of fibre-rich food such as wholegrains and vegetables.
• Reduce intake of saturated fat.

## Follow-up

HbA_1C_ should be monitored at least annually. If glycaemia deteriorates despite lifestyle intervention, metformin may be indicated following appropriate counselling and determination that renal function is sufficient.[5] Lifestyle advice should be continued. Both HbA1c/FBG and renal function should be monitored if metformin is started, and the drug should be discontinued if there is no impact on HbA1c/FBG within 6–12 months, or if renal function deteriorates.[5] Metformin may also be suitable for those unable to participate in lifestyle interventions.

## Author contributions

LH wrote the first draft of the article. All authors contributed to and approved the final version.

## Disclosure statement

We have read and understood the LJPC policy on declaration of interests and declare the following interests: AM has received MRC funding (to the institution) for a trial relating to intermediate hyperglycaemia.

## Why this matters to me

Despite well-demonstrated efficacy under trial conditions, we are concerned about the lack of demonstrated effectiveness of lifestyle interventions for diabetes prevention. As population-level impact will be influenced by identification of relevant patients and appropriate counselling, we feel it is important that information about the NHS Diabetes Prevention Programme is disseminated widely. This should also help improve associated routine primary care data collection, which may be important as NHS England have published little information about intended programme evaluation. We are also keen to emphasise that lifestyle interventions will not be successful in all patients, and that follow-up (including to prevent potential disengagement with lifestyle change if the programme is unsuccessful), attention to additional cardiovascular disease risk factors, and support for additional approaches to the current diabetes epidemic, will be important.

## Key message

Timely recognition and appropriate management of those at high risk of diabetes could help mitigate the impact of this epidemic.

## Related LJPC papers

Zaineb Amin Mohsin, Anna Paul & Senan Devendra (2015) Pitfalls of using HbA1C in the diagnosis and monitoring of diabetes, London Journal of Primary Care, 7:4, 66–69. http://dx.doi.org/10.1080/17571472.2015.11493437

Edouard Mills & Senan Devendra (2015) Steroid-induced hyperglycaemia in primary care, London Journal of Primary Care, 7:5, 103–106. http://dx.doi.org/10.1080/17571472.2015.1082344

## Governance

Department of Primary Care and Public Health, Imperial College London, UK
